# Diagnostics in Waldenström’s macroglobulinemia: a consensus statement of the European Consortium for Waldenström’s Macroglobulinemia

**DOI:** 10.1038/s41375-022-01762-3

**Published:** 2022-11-26

**Authors:** Irene Dogliotti, Cristina Jiménez, Marzia Varettoni, Dipti Talaulikar, Tina Bagratuni, Martina Ferrante, José Pérez, Daniela Drandi, Noemí Puig, Milena Gilestro, María García-Álvarez, Roger Owen, Wojciech Jurczak, Alessandra Tedeschi, Veronique Leblond, Efstathios Kastritis, Marie José Kersten, Shirley D’Sa, Michal Kaščák, Wolfgang Willenbacher, Aldo M. Roccaro, Stephanie Poulain, Pierre Morel, Charalampia Kyriakou, Falko Fend, Josephine M. I. Vos, Meletios A. Dimopoulos, Christian Buske, Simone Ferrero, Ramón García-Sanz

**Affiliations:** 1 Unit of Hematology, Department of Biotechnology and Health Sciences, University of Torino, Torino, Italy; 2 Hematology Department, University Hospital of Salamanca, Research Biomedical Institute of Salamanca (IBSAL), CIBERONC and Center for Cancer Research-IBMCC (University of Salamanca-CSIC), Salamanca, Spain; 3 Division of Hematology, Fondazione IRCCS Policlinico San Matteo, Pavia, Italy; 4 Canberra Health Services, College of Medicine, Biology and Environment Australian National University, Canberra ACT, Australia; 5 Department of Clinical Therapeutics, School of Medicine, National and Kapodistrian University of Athens, Athens, Greece; 6 The Leeds Teaching Hospitals National Health Service Trust, Leeds, UK; 7 Maria Sklodowska-Curie National Research Institute of Oncology, Krakow, Poland; 8 ASST Grande Ospedale Metropolitano Niguarda Hospital, Milan, Italy; 9 Département d’Hématologie Hôpital Pitié-Salpêtrière APHP, UPMC Université Paris, Paris, France; 10 National and Kapodistrian University of Athens School of Medicine, Athens, Greece; 11 Department of Hematology, Amsterdam UMC, Location University of Amsterdam, Cancer Center Amsterdam and LYMMCARE (Lymphoma and Myeloma Center Amsterdam), Amsterdam, The Netherlands; 12 Centre for Waldenströms Macroglobulinaemia and Related Conditions, University College London Hospitals National Health Service Foundation Trust, London, UK; 13 Department of Haematooncology, University Hospital Ostrava and Faculty of Medicine, University of Ostrava, Ostrava, Czech Republic; 14 Department of Haematology and Oncology, Internal Medicine V, Innsbruck University Hospital & Syndena GmbH, Connect to Cure, Innsbruck, Austria; 15 Clinical Research Development and Phase I Unit, ASST Spedali Civili di Brescia, Brescia, Italy; 16 Laboratory of Hematology, Biology and Pathology Center, CHU of Lille, INSERM UMR-S 1277, Team 4, Oncolille, Lille, France; 17 Service d’Hematologie Clinique et Therapie Cellulaire, Centre Hospitalier Universitaire d’Amiens-Picardie, Amiens, France; 18 Institute of Pathology and Comprehensive Cancer Centre, Eberhard-Karls-University, University Hospital Tübingen, Tübingen, Germany; 19 Institute of Experimental Cancer Research, Comprehensive Cancer Center Ulm, University Hospital of Ulm, Ulm, Germany; 20 Hematology Division 1U, “AOU Città della Salute e della Scienza di Torino”, Torino, Italy

**Keywords:** B-cell lymphoma, Genetic testing

## Abstract

The diagnosis of Waldenström’s macroglobulinemia (WM), an IgM-associated lymphoplasmacytic lymphoma, can be challenging due to the different forms of disease presentation. Furthermore, in recent years, WM has witnessed remarkable progress on the diagnostic front, as well as a deeper understanding of the disease biology, which has affected clinical practice. This, together with the increasing variety of tools and techniques available, makes it necessary to have a practical guidance for clinicians to perform the initial evaluation of patients with WM. In this paper, we present the consensus recommendations and laboratory requirements for the diagnosis of WM developed by the European Consortium of Waldenström’s Macroglobulinemia (ECWM), for both clinical practice as well as the research/academical setting. We provide the procedures for multiparametric flow cytometry, fluorescence in situ hybridization and molecular tests, and with this offer guidance for a standardized diagnostic work-up and methodological workflow of patients with IgM monoclonal gammopathy of uncertain significance, asymptomatic and symptomatic WM.

## Introduction

The Consensus Panel Recommendations from the Second International Workshop on Waldenström’s Macroglobulinemia (WM) [1] state that the diagnosis of WM requires the following clinical and pathological criteria: presence of infiltration of clonal lymphoplasmacytoid cells documented by bone marrow (BM) biopsy (lymphoplasmacytic lymphoma (LPL)) and presence of monoclonal IgM in the serum, irrespective of the M-protein size. The 2016 WHO classification defines lgM monoclonal gammopathy of undetermined significance (IgM-MGUS) by the presence of a serum lgM paraprotein below 30 g/l, BM lymphoplasmacytic infiltration <10%, and no evidence of end-organ damage related to the underlying lymphoproliferative disorder [2]. Recent updates of this classification have not changed this view [3, 4], although they have stressed the importance of the mutational landscape in WM. Therefore, a BM biopsy remains mandatory for the differential diagnosis between WM, IgM-MGUS and other B-cell lymphoproliferative disorders (B-LPDs) (Table 1) [5, 6]. In addition, although not yet recognized by the WHO classification, there are some patients with clinical features attributable to IgM monoclonal protein but no evidence of lymphoma (IgM-related disorders) who should also be considered for BM evaluation to rule out a WM [1].

**Table 1:** Classification of Waldenström’s macroglobulinemia and related disorders.

	IgM monoclonal protein^a^	Bone marrow infiltration^b^	Symptoms attributable to IgM	Symptoms due to tumor infiltration^c^
Symptomatic WM	+	+	+^d^	+^d^
Asymptomatic WM	+	+	−	−
IgM-related disorders^e^	+	−	+	−
IgM-MGUS	+	−	−	−

*IgM-MGUS* IgM monoclonal gammopathy of uncertain significance, *WM* Waldenström’s macroglobulinemia.

^a^The panel considered to be inappropriate to define an IgM concentration to distinguish MGUS from WM. However, it should be noted that IgM concentration rarely, if ever, exceeds 30 g/dl in MGUS.

^b^Patients with unequivocal BM infiltration by lymphoplasmacytic lymphoma will be considered to have WM, while patients without evidence of infiltration will be considered to have MGUS. However, it is acknowledged that in some patients, equivocal evidence of BM infiltration is demonstrable. This may be manifested in several ways including the presence of clonal B-cells by flow cytometry or PCR in the absence of morphological evidence of BM infiltration. Alternatively, patients may have equivocal bone marrow infiltrates without confirmatory phenotypic studies. It is considered that these patients should be classified as MGUS until further data become available. According to the 2016 WHO classification, bone marrow lymphoplasmacytic infiltration is <10% in IgM-MGUS.

^c^Symptoms attributable to tumor infiltration will include any of the following manifestations: constitutional symptoms, cytopenia(s), or organomegaly.

^d^It is required the presence of one or both groups of symptoms.

^e^It is well recognized that a population of patients exist who have symptoms attributable to the IgM monoclonal protein but no overt evidence of lymphoma. Such patients may present with symptomatic cryoglobulinemia, amyloidosis, or autoimmune phenomena such as peripheral and cold agglutinin disease. It is appropriate to consider these patients as a clinically distinct group and the term “IgM-related disorders” is proposed. Adapted from Owen et al. [1].

Multiparametric flow cytometry (MFC) and molecular techniques may help to confirm the diagnosis, especially to discriminate WM from other IgM-secreting disorders. MFC analysis has been shown to accurately quantify the number of clonal cells, although it may underestimate the amount of marrow infiltration compared to the BM biopsy [7], probably due to the hemodilution effect during BM aspiration. A progressive increase in the number of light-chain-isotype-positive B-cells from IgM-MGUS to smoldering and to symptomatic WM has been demonstrated [8]. However, the pattern of antigen expression and the relative fractions of individual marker expressing clonal B-cells remain stable during disease progression [9].

## Main biological characteristics in WM

Important advances in understanding the biology of WM have been made in recent years, leading to an increased toolset for differential diagnosis. Using whole genome sequencing, Treon et al. [10] identified *MYD88*^*L265P*^ as a highly recurrent (~95%) somatic mutation in patients with WM. Several studies using different techniques, such as Sanger sequencing, and allele-specific quantitative PCR (ASqPCR) [11–14], confirmed that *MYD88*^*L265P*^ is present in >90% of WM, whereas it is absent in patients with multiple myeloma (MM) (including IgM isotype) [15], and less frequently found in marginal zone lymphoma (MZL) with plasmacytic differentiation or chronic lymphocytic leukemia (CLL) [16, 17]. Therefore, *MYD88*^*L265P*^ assessment is considered crucial to discriminate between WM and other B-LPDs with overlapping clinical features. Mutations in the *CXCR4* gene were identified as the second most common alterations in WM (30–40% patients) [18, 19], and play an important role in WM pathogenesis and disease progression [20, 21]. These mutations might also impact the clinical presentation and outcome of WM patients. Thus, *MYD88*^*L265P*^/*CXCR4*^*WHIM*^ patients may present with a more aggressive clinical behavior, and inferior response to Bruton Tyrosine Kinase inhibitors (BTKi) [22]. On the other hand, higher risk of transformation to aggressive lymphoma and shorter overall survival were reported in *MYD88* wild-type cases [23].

WM presents with a median of 2–3 chromosomal abnormalities per patient [24]. Deletion of 6q (−6q or del6q) is the most frequent chromosomal abnormality (40–50% of patients) [25] and it is directly related with progression from asymptomatic to symptomatic WM [26]. Deletion of 17p/*TP53* is present in 8–15% of WM patients, and *TP53* mutations are present in a small subset of patients with poor prognosis [27–30].

Asymptomatic patients with IgM monoclonal component below 1.5 g/dl (or 15 g/l) and normal serum free light-chain ratio have a very low-risk of progression to overt WM or other lymphoproliferative malignancies, and BM biopsy is not generally recommended at this stage, outside the context of clinical trials, and in the absence of any potential IgM-related symptom [31, 32]. However, the cut-off point of 1.5 g/dl could be misleading since in WM, there is no concordance between BM infiltration, IgM levels and patient symptoms. Thus, patients with predominant lymphocytic infiltration and poor plasmacytic differentiation may have low serum IgM levels [8, 33] and could be incorrectly classified as MGUS without a BM evaluation [34]. Consequently, although the value of BM assessment in asymptomatic individuals is not fully established, most groups currently agree that it may provide prognostic information about the risk of progression and the indication of the BM biopsy should be discussed [6].

Since WM is a rare disease and procedures may vary across different laboratories, we aim to provide consensus recommendations of the European Consortium of Waldenström’s Macroglobulinemia (ECWM) on diagnostics in this lymphoma subtype [6, 35]. We will discuss the basic and essential procedures that must be performed by local centers for the diagnosis and initial evaluation of WM patients, as well as more complex techniques that should be considered for precise pre-treatment evaluation, disease monitoring and research studies to be carried out in referral centers. In addition, detailed procedures for MFC, fluorescence in situ hybridization (FISH) and molecular tests will be provided.

ECWM-supported recommendations were made based on an international consensus reached through a Delphi survey, with two rounds of open discussion and a virtual consensus meeting; the ECWM is composed of hematologists, pathologists, and biologists/researchers in the field of WM, and all authors participated in the process. A first draft was prepared by the first, senior and corresponding authors following the usual procedures and comments made at the ECWM meetings; the initial versions were distributed to all authors with two rounds of open discussions and comments. Once the main draft was agreed, the most debated points were selected to develop 14 recommendations (10 for diagnostic purposes and 4 for helping in research), and a Delphi survey was launched among the authors. The Delphi score range from 1, completely disagree, to 9, completely agree. Nine questions were approved in a first survey round, based on a 75% agreement of 8–9, or a 90% agreement of 6–9. The five remaining recommendations were re-written considering the opinion of the dissenting authors. A second round was sufficient to reach the final consensus presented in the following paragraphs. There was an initial unanimity on 8 of the questions, and on the final 14 recommendations. The consensus recommendations represent the views of the panel and are potentially applicable to both clinical practice and biologic studies in the context of clinical trials. Future evidence might lead to updates in this guidance, which is now intended to provide a robust framework to support clinicians and avoid discrepancies in the diagnosis of WM.

## Essential diagnostic requirements for WM

Although WM can present as an asymptomatic entity, most patients initially consult due to B-symptoms, such as fevers, night sweats or unintentional weight loss. Other common symptoms include fatigue, malaise, and shortness of breath, usually due to anemia, and increased bleeding or bruising that can be associated with thrombocytopenia or acquired von Willebrand disease [5]. Finally, the third group of frequent symptoms are associated with hyperviscosity, including epistaxis, headache, blurred vision, vertigo, and tinnitus. Other symptoms can be present, but a comprehensive review exceeds the intent of the present working consensus, which focuses on the laboratory steps that should follow the identification of the IgM monoclonal protein and/or the initial symptoms mentioned above.

### Essential laboratory analyses in WM: general recommendations

The quality and quantity of the material required for WM diagnosis are critical. Currently, both BM and peripheral blood (PB) samples are helpful, while other tissues (lymph node, pleural effusion, cerebrospinal fluid (CSF)) may be useful to further characterize the disease. However, WM diagnostic criteria still require an histological evaluation of the BM biopsy for the final diagnosis [5]. Newer, patient-friendly molecular tools applicable to PB samples might be preferred over classical BM biopsy, but they are not yet sufficiently evaluated and standardized to provide a definitive diagnosis.

A relatively large amount of BM is needed to perform (at least) MFC and molecular studies, and during aspiration of such a volume, there is a significant risk of hemodilution. Clots may also affect the quality of samples, especially in patients with marked hyperviscosity syndrome, cold agglutinins or cryoglobulinemia. No recommendations are yet available for optimization of marrow aspiration, although normalization is an option when using MFC [36]. Clots from the BM biopsy may provide pathological material for molecular studies.

Optimally, the timing of marrow aspiration should allow rapid processing of the sample in the local laboratory or rapid shipment to a central laboratory. The central laboratory for each local site should be assigned based primarily on geographic criteria. Samples must be stored at 4 °C when long shipping times are anticipated (>48 h), while room temperature storage can be considered when samples are delivered to the central laboratory in a short time (up to 24 h). Alternatively, samples can be collected in specialized Cell-Free DNA BCT tubes (©Streck) to ensure genomic DNA stability up to 14 days.

### Sample types and processing protocols: technical aspects

#### Bone marrow biopsy

A BM trephine biopsy with a minimal length of 20 mm containing marrow spaces is considered adequate for the histopathological diagnosis of WM/LPL. Formalin fixation and decalcification by EDTA provide the best results for morphological, immunohistochemical and molecular examination, while alternative fixatives and acid decalcification can severely compromise antigen expression and preservation of DNA and RNA. The infiltration pattern is usually divided into 3 to 4 types, including nodular, para-trabecular, (interstitial), and diffuse. According to several reports, para-trabecular invasion pattern is one of the pathological features of WM and can be useful for differentiation from MZL [1, 35, 37]. It is also important to evaluate and exonerate the presence of other BM diseases, e.g., myelodysplastic syndrome.

In addition to standard hematoxylin and eosin and/or Giemsa stains and a reticulin stain for the assessment of fibrosis, immunohistochemical stains are used for the characterization and quantification of the infiltrate [35]. The pan-B-cell marker CD20 (alternatively CD79a, which also stains the plasma cell (PC) component, or PAX5) and a minimal antibody panel containing IgM and immunoglobulin light chains should be used to demonstrate light-chain restriction [5, 6]. Markers such as CD38 (which also stains lymphocytes) and CD138 can be used to evaluate the degree of plasmacytic differentiation. Additional markers to exclude other B-cell lymphomas and MM should be included as deemed necessary depending on the availability of flow cytometric phenotyping. Although WM has a non-specific immunophenotype, CD5, CD23, CD10, cyclin D1, LEF1, and CD56 staining is helpful in excluding most differential diagnoses. However, discrimination from splenic MZL may be difficult. Presence of plasmacytoid differentiation, monoclonal PC, and increased mast cells are more suggestive of WM than MZL [37]. EDTA-decalcified BM trephines may serve as an excellent alternative source for detection of *MYD88* and *CXCR4* mutations by ASqPCR or sequencing [38]. In those patients with symptoms related with cardiac, renal or neurological dysfunctions, a specific search for amyloid deposits should be performed [39].

#### Bone marrow aspiration

During BM aspiration, representative samples should be collected for a correct initial cytomorphological evaluation of lymphoplasmacytic and PC. For standard baseline analyses, samples should be collected in at least three EDTA tubes for MFC and molecular analyses, and one sodium heparin tube for FISH purposes. The median percentage of tumor cells in BM samples of MGUS, asymptomatic and symptomatic WM is 2.2%, 8.7%, and 12.2%, respectively [8]. For these numbers, FISH studies are below the sensitivity threshold. Therefore, enrichment of BM samples for CD19+ cells by immunomagnetic approaches would be advisable [26]. This option cannot be mandatory for local centers but should be for referral centers and requires the third additional EDTA tube. Adequate cellularity of the sample is also key to perform reliable BM analyses. Therefore, the collection of 12–20 ml divided into 4 tubes (3 EDTA and 1 sodium heparin) is recommended since a standard procedure requires >3 × 10^7^ cells.

#### Peripheral blood

When BM samples are difficult to obtain, in rare cases of leukemic WM, PB samples can be used as an alternative for diagnostic procedures. Whole blood cells can be used for MFC analysis and genomic studies. EDTA samples are preferred (2 tubes of 10 ml), as they allow for CD19+ cell enrichment, improving throughput and accuracy [40].

In case PB samples are used for circulating tumor DNA (ctDNA) analysis, it is important to preserve the integrity of the circulating nucleic acid; consequently, PB EDTA samples must arrive at the laboratory within 4 h after the extraction. Alternatively, ctDNA can be collected in ©Streck tubes (preferably, 2 tubes of 10 ml), specifically designed for shipment to central laboratories (Supplementary Information, Appendices A, B and D).

#### Cerebrospinal fluid

For cases of suspected Bing Neel Syndrome, CSF analysis should include assessment of cytology, flow cytometry, *MYD88* testing, and immunoglobulin gene rearrangement analysis, along with routine biochemistry and leukocyte cell count by MFC [41–43]. Simultaneous *MYD88* testing in PB or flow cytometric red cell quantification must be performed to identify a possible contamination of CSF with PB during lumbar puncture. This analysis should be carried out by experienced laboratories, so centralization is recommended. *CXCR4* mutations have not been identified in CSF, probably due to the relatively small number of cells, which makes it difficult to obtain sufficient DNA for sequencing. Digital PCR (dPCR) for *MYD88*^*L265P*^ detection in ctDNA from CSF is a highly sensitive method [44, 45], but requires further investigation before it can be implemented in routine clinical practice. Details on sample collection, storage and shipment are available in Appendices A and B (Supplementary Information).

### Multiparametric flow cytometry protocols

Approximately 4 ml of EDTA-anticoagulated BM-aspirated sample is needed to perform an immunophenotypic analysis using an 8–12 color direct immunofluorescence stain and a lysis technique, with different combinations of monoclonal antibodies: e.g., Pacific Blue [PacB]/Pacific Orange [PacO]/fluorescein isothiocyanate [FITC]/phycoerythrin [PE]/peridinin-chlorophyll protein-cyanin 5.5 [PerCP-Cy5.5]/PE-cyanin 7 [PE-Cy7]/allophycocyanin [APC]/APCH7 (Table 2) [46].

**Table 2:** Minimum panels for cell characterization by flow cytometry of Waldenström’s macroglobulinemia at diagnosis.

Tube	FITC	PE	PERCP-Cy5.5	PE-Cy7	APC	APC-H7/APC-Cy7	PacB/BV450	PacO/OC515
1	SIgM (5 µl)	CD25 (10 µl)	CD22 (10 µl)	CD19 (5 µl)	CD27 (5 µl)	CD38 (3 µl)	CD20 (5 µl)	CD45 (5 µl)
2	CyIgM (5 µl)	CyL (5 µl)	CD5 (10 µl)	CD19 (5 µl)	CyK (5 µl)	CD38 (3 µl)	CD20 (5 µl)	CD45 (5 µl)

Adapted from Puig et al. [33].

*FICT* fluorescein isothiocyanate, *PE* phycoerythrin, *PERCP* peridinin-chlorophyll-protein complex, *Cy5.5* Cyanine 5, *Cy7* Cyanine 7, *APC* allophycocyanin, *PacB* Pacific Blue, *BV450* Brilliant Violet 450, *PacO* Pacific Orange, *OC515* Orange Cytognos 51.

Pre-analytical procedures are important in the evaluation of suspected WM, as the quality of the BM aspirate affects the MFC results; therefore, some authors suggest using the first aliquot of the BM sample (i.e., the “first draw”) for MFC analysis to reduce the hemodilution. Precise evaluation of the BM aspirate by MFC is necessary to determine the quality of the sample, particularly in cases with low disease burden [36]. Some approaches to normalize the sample against hemodilution can also be used [47].

The characteristic immunophenotypic features of WM clonal B-cells are intracytoplasmic and surface light-chain restriction, as well as surface expression of pan-B-cell antigens (CD19, CD20), together with CD22^+^dim, CD25+, CD27+ and IgM+; other antigens such as FMC7, BCL2, PAX5, CD81 and CD79b are usually positive as well, while CD10, CD11c, CD103 and CD23 are mostly absent. CD5 is expressed in 5–20% of cases [48]. CD27 and CD200 frequently show heterogeneous bimodal patterns of expression. CD305 (LAIR1) is particularly useful to detect light-chain restricted clonal B-cells due to its homogenous lack of expression in 69% of WM cases, which contrasts to the bimodal heterogeneous staining in normal B-cells [9]. WM and MZL can have an overlapping phenotypic profile, although WM usually has homogeneous expression of CD25 and weak expression of CD22 (~90% of cases), whereas MZL is usually CD22+ + CD25− (~80%) [46]. In addition, CD27 expression is usually higher in MZL. This pattern together with the histological characteristics (i.e., pattern of BM infiltration, dendritic meshwork, sinusoidal localization, mast cell presence), clinical characteristics and molecular results can help to differentiate between the two entities [37].

Among total BM nucleated cells, PC percentages are not very different among IgM-MGUS and WM patients; by contrast, a progressively higher percentage of light-chain-isotype PC is noted from IgM-MGUS to smoldering and to symptomatic WM [8]. PC are also clonally restricted, and express CD38, CD138, variable CD45, CD79A, and low levels of CD19 and CD20. They consistently lack CD56 expression, which together with CD19 expression, can be reliably used to differentiate clonal PC in WM from the clonal PC infiltrate observed in MM (usually positive for CD56 and negative for CD45 and CD19). Furthermore, the PC count can also be helpful in discriminating between WM and MZL, because the presence of clonal PC is common in WM and rare in MZL [9, 37]. According to recent findings, a correct assessment of clonal PC in WM might be important, both for the correlation with the amount of IgM paraprotein at clinical presentation, and for the potential role as predictive biomarkers of treatment response, although this should be validated in larger and prospective studies [33, 49].

For screening purposes, BM samples from patients with an IgM monoclonal gammopathy should preferably be processed following the general recommendations of the EuroFlow group (Supplementary Information, Appendix C) [36].

Most of these studies can be performed in local laboratories but quick shipment and centralization are recommended if a correct procedure cannot be warranted.

### Genetic analysis

For a complete mutational analysis in WM, it is preferable to send the samples to a reference laboratory to ensure adequate sensitivity and reproducibility. However, it is also recommended that non-referral laboratories can perform *MYD88* mutation screening on BM samples for diagnostic purposes. PCR methods following the operating procedures described in the Supplementary Information are recommended, and a detection limit of at least 1% is mandatory.

Non-L265P *MYD88* mutations have also been identified in patients with WM, including S219C, M232T, and S243N [18]. Next generation sequencing (NGS) and Sanger sequencing of BM samples can be used to detect the *MYD88* mutations outside the L265P site, but NGS is not widespread yet and Sanger sequencing usually does not have optimal sensitivity, especially for samples not enriched in CD19+ cells [50]. In addition, the application of these tools can be limited by their turn-around time, cost, quality of the sample, and BM infiltration. PCR analysis (ASqPCR [11, 12, 51], dPCR [44]) is preferred over sequencing techniques for *MYD88*^*L265P*^ detection because of its higher sensitivity and faster turn-around time with lower costs.

## Additional requirements for reference laboratories

### Cytogenetics and FISH analysis

The role of conventional cytogenetics in WM is not well defined; therefore, standard karyotyping is not recommended for these patients although it may help in the differential diagnosis, especially in cases without the *MYD88*^*L265P*^ mutation. FISH studies in CD19+ cells are well feasible. It is recommended to perform analysis of del6q and del17p at least in the central labs. Alternative methods, such as SNP arrays [52] or whole genome sequencing [18] in samples with CD19+ enrichment may also be used, but cannot yet be considered for daily laboratory practice.

### Mutational studies

#### MYD88^L265P^ detection

The main requirement for central laboratories is to be able to analyze *MYD88*^*L265P*^ by molecular techniques with a detection limit of at least 1 × 10^−3^. The accepted techniques in terms of reproducibility and sensitivity include ASqPCR and dPCR on unselected BM samples, as well as Sanger or NGS on selected BM CD19+ cells [11–14, 44, 53]. Alternatively, BM trephines may be used for mutational screening, particularly in samples with high percentage of infiltrating tumor cells [54], being especially useful when the analysis has not been performed on fresh BM aspirate samples.

For patients that are negative for *MYD88*^*L265P*^ by ASqPCR, complete gene sequencing should be performed looking for non-L265P mutations [18]. For these purposes, only Sanger or NGS in samples enriched for CD19+ cells can provide reliable results. When the *MYD88* gene is in a full germline configuration, another diagnosis should be considered, from MZL to IgM-MM. This is also relevant when mutational analysis is used for therapeutical decision-making, as BTKi work in patients with (rare) non-L265P mutations, as well as in cold agglutinin disease, which is non-L265P mutated [55, 56].

#### CXCR4^WHIM^ detection

Central laboratories are also required to provide the option to detect *CXCR4* mutations. Originally, this analysis was not recommended at initial diagnosis for all WM patients, but its importance is increasing rapidly, due to the widespread use of BTKi in clinical practice. Therefore, most authors suggest performing a *CXCR4* mutational screening before BTKi treatment or in case of poor response or progression, and, if possible, at initial diagnosis as well.

Although many laboratories routinely investigate only the most common variant of *CXCR4*, *CXCR4*^*S338X*^, present in nearly half of cases [20], it is important to note that there are >40 different mutations. Consequently, mutational analyses of *CXCR4* should be performed by Sanger sequencing or NGS on CD19+ enriched BM samples. In addition and in contrast to *MYD88*^*L265P*^ mutation, *CXCR4* mutations are frequently sub-clonal [18, 57].

#### Other targets: investigational molecular tools

An attractive new tool for molecular studies in WM is the so called “liquid biopsy”, that is, the detection of ctDNA in plasma or other biological fluids. It is a less invasive, patient-friendly test that could provide a good diagnostic yield, even comparable to BM, and might allow serial mutational studies without the need for repeated BM aspirates [44, 53]. In addition, ctDNA analysis can be representative of extra-medullary disease and of the whole marrow compartment, making it a potentially cost-effective approach that avoids BM aspiration sampling bias.

Detection of *MYD88* and, more recently, *CXCR4* somatic mutations in ctDNA from PB of WM patients is an area under development, with initial promising results, showing a high concordance with tumor burden [53]. Recently, the introduction of dPCR has shown several practical advantages over qPCR, being particularly useful for ctDNA minimal residual disease studies [58, 59].

The newly introduced Competitive Allele-Specific TaqMan® PCR (Cast-PCR) technology is highly specific, sensitive and can detect small amounts of mutated DNA in samples with large amounts of wild-type DNA. It has already been tested to detect the *MYD88*^*L265P*^ mutation in both tumor-derived DNA and ctDNA, showing a sensitivity of 10^−3^, with the possibility of using very low amounts of DNA (as low as 20 pg) [60].

However, all these techniques need to be standardized and implemented in prospective studies before they can be used in clinical practice, and the current recommendation to perform BM aspiration for MFC and molecular analyses would probably be maintained.

The analysis of *TP53* mutations in CD19+ sorted cells is also being investigated, as is being done in CLL by Sanger sequencing or TP53-specific NGS approaches. The analysis of other mutations (Table 3) is also a possibility that could be considered by the physician.

**Table 3:** Frequency of somatic mutations occurring in Waldenström’s macroglobulinemia as a basis for the design of a next-generation sequencing panel.

	Hunter et al. [18]	Poulain et al. [57]	Varettoni et al. [30]	Jiménez et al. [19]	All
Gene	*N* = 30	*N* = 98	*N* = 62	*N* = 47	*N* = 237
*MYD88*	27	77	53	46	86%
*CXCR4*	27	24	14	17	35%
*KTM2D*	2	NA	15	NA	18%
*TP53*	2	9	6	1	7.6%
*CD79A/B*	2	9	2	4	7.2%
*ARID1A*	5	NA	3	2	7.2%
*NOTCH2*	1	NA	3	NA	4.3%
*PRDM1*	0	NA	4	NA	4.3%
*HIST1H1E*	0	NA	NA	3	3.8%
*MYBBP1A*	2	NA	0	2	2.9%
*TRAF3*	1	NA	1	2	2.9%
*TRAF2*	1	NA	NA	1	2.6%
*RAG2*	1	NA	NA	1	2.6%
*HIST1H1B*	0	NA	NA	1	1.3%
*HIST1H1D*	0	NA	NA	1	1.3%

*NA* not applicable.

In conclusion, accepted samples for mutation detection and preferred techniques can be summarized as follows:

- Unsorted BM (either white blood cells or mononuclear cells) might be analyzed by ASqPCR, although dPCR is preferred.
- In case of BM biopsy, EDTA-decalcified, paraffin-embedded BM trephines or paraffin-embedded BM clots (non-decalcified) can be used for ASqPCR/dPCR or NGS (except in cases of minimal BM infiltration).
- PB is suboptimal for mutational analysis especially by ASqPCR; therefore, PB samples should be analyzed by dPCR to increase sensitivity.
- For IgM-MGUS and follow up samples with low tumor burden, dPCR is preferable to ASqPCR.
- CD19+ sorted BM cells can be analyzed by Sanger sequencing and NGS. Although CD19+ selection is not mandatory, it helps to increase the sensitivity of the assays and is recommended when available.
- Plasma (ctDNA) should be analyzed by dPCR. For plasma selection, PB must be collected in EDTA tubes, if processed within 4 h from drawing, or in ©Streck tubes, if processed after 4 h from drawing (Appendix A, Supplementary Information). This method is still under research.
- In case Bing Neel syndrome is suspected, *MYD88*^*L265P*^ analysis on CSF should be carried out by highly sensitive methods.

Operative procedures for mutational screening can be found in the Supplementary Information.

## Summary of recommendations for WM laboratory diagnosis

The work-up of suspected newly diagnosed WM patients should include pathological, MFC, and molecular studies guided by the recommendations shown in Table 4. All centers should warrant the studies shown in this table, locally or by shipment to referral centers.

**Table 4:** Recommendations for the work-up of suspected newly diagnosed Waldenström’s macroglobulinemia patients.

**Diagnostic studies**
(1) BM biopsy is mandatory for a correct diagnosis to distinguish between the different forms of IgM monoclonal gammopathies, and it is particularly informative in distinguishing between WM and *MYD88*^*L265P*^ MZL cases with IgM monoclonal protein
(2) BM aspiration samples have to be sufficient, both in terms of quantity and quality, to perform MFC, cytogenetic and molecular studies in both unselected and CD19+ enriched samples. This implies sampling up to 12–20 ml of BM distributed in 3 EDTA tubes (flow cytometry, molecular and cell enrichment) and 1 sodium heparin tube (FISH studies)
(3) MFC studies have to be done in the non-fractionated BM aspiration to distinguish and assess the size of the tumor clone and exclude other B-LPDs and plasma cell dyscrasias. To do so, both the lymphoplasmacytic and plasma cell compartments should be characterized and counted
(4) *MYD88*^*L265P*^ assessment is mandatory during the diagnostic work-up of WM and related disorders, and must be done in BM samples, using ASqPCR or dPCR
(5) *MYD88*^*L265P*^ molecular analysis on CD19+ enriched samples is not mandatory but is required if using low sensitivity techniques (i.e., Sanger sequencing) or for samples with low infiltration
(6) *MYD88*^*L265P*^ assessment in DNA from PB alone is not sufficient for a correct diagnosis due to frequent false negative results, even if the sample has been enriched in CD19+ cells, or ctDNA was used, and it is therefore not recommended
(7) In patients with a high probability of WM diagnosis but no detection of *MYD88*^*L265P*^ in the BM with reliable sensitive methods, it is highly recommended to do complete *MYD88* gene sequencing by Sanger or NGS, especially if BTKi are planned to be used. Sample must be enriched in CD19+ cells by immunomagnetic methods or flow cytometry sorting^a^
(8) *CXCR4*^*WHIM*^ analysis is not currently considered mandatory in all WM patients outside clinical trials. However, if the patient is going to be treated with BTKi, it is highly recommended to perform this analysis to unveil treatment resistance mechanisms and anticipate slow responses. Analysis should be done by Sanger or NGS, using genomic DNA from BM cells after CD19+ enrichment. If CD19+ cell enrichment is not feasible, more sensitive approaches such as ASqPCR or dPCR on unsorted samples must be done, considering that these methodologies do not cover all mutations^a^
(9) FISH studies with suitable probes for 6q21–25 and TP53/17p should be carried out by local or reference laboratories in BM samples enriched in CD19+ cells^a^
(10) Alternative samples from other tissues (PB, lymph nodes, CSF, pleural or peritoneal effusions) can be used for additional diagnostic characterization if indicated. In this case, the presence of sufficient tumor cells in the cellular suspensions must be warranted by flow cytometry
**Research studies**
(11) New promising approaches with good sensitivity (Cast-PCR, high resolution melting analysis, dPCR, etc.) could be used as alternatives for detecting the *MYD88*^*L265P*^ mutation and *CXCR4* mutations. However, they still have to be considered as investigational and require validation before they could be considered reliable for diagnostic purposes
(12) *MYD88*^*L265P*^ assessment in DNA from PB could be of help in the diagnosis of WM, especially when the sample has been enriched in CD19+ cells, or when ctDNA is used for the study. However, the real feasibility of this approach remains to be assessed and should be investigated, especially in the context of clinical trials
(13) Although PB studies are a good addition to the diagnostic work-up, their exclusive use, without BM evaluation, could be of value in low-risk IgM-MGUS (monoclonal protein <15 g/l, normal free light-chain ratio). However, they should still be considered under the umbrella of research studies^a^
(14) Although *TP53* mutation analysis is of high interest in WM due to prognostic and potential therapeutic implications, especially when immunochemotherapy is planned, it should be considered investigational at this moment. This analysis can be performed by reference laboratories using Sanger sequencing or NGS, using genomic DNA from BM cells after CD19+ cell enrichment^a^

*ASqPCR* allele-specific quantitative PCR, *BM* bone marrow, *BTKi* Bruton’s tyrosine kinase inhibitors, *B-LPDs* B-cell lymphoproliferative disorders, *Cast-PCR* Competitive Allele-Specific TaqMan® PCR, *CSF* cerebrospinal fluid, *ctDNA* circulating tumor DNA, *dPCR* digital PCR, *FISH* fluorescence in situ hybridization, *IgM-MGUS* IgM monoclonal gammopathy of uncertain significance, *MFC* multiparametric flow cytometry, *MZL* marginal zone lymphoma, *NGS* next-generation sequencing, *PB* peripheral blood, *WM* Waldenström’s macroglobulinemia.

^a^Statements that required a second round.

## Supplementary information

Supplementary information
