# Supplementary material for: Diagnostics in Waldenström’s macroglobulinemia: a consensus statement of the European Consortium for Waldenström’s Macroglobulinemia
Source: Leukemia. 2022 Nov 26;37(2):388–95. doi: 10.1038/s41375-022-01762-3 (PMC9898035; doi:10.1038/s41375-022-01762-3)
Supplement: Supplementary file 1 — Supplementary information [file 41375_2022_1762_MOESM1_ESM.docx]

**SUPPLEMENTARY INFORMATION**

APPENDIX A - Operative considerations for peripheral blood samples collection and shipment for circulating tumor DNA evaluation

Peripheral blood (PB) samples for molecular analyses on circulating tumor DNA (ctDNA) of diagnostic materials should be collected in 2 Cell-Free DNA BCT tubes (©Streck).

**20 mL of PB** should be collected in 2 Cell-Free DNA BCT tubes (©Streck, 10 mL each).

Cell-Free DNA BCT is a direct draw whole blood collection tube intended for collection, stabilization, and transportation of plasmatic ctDNA. The formaldehyde-free preservative reagent contained in Cell-Free DNA BCT stabilizes nucleated blood cells, preventing the release of cellular genomic DNA, and inhibits nuclease-mediated degradation of ctDNA, contributing to the overall stabilization of ctDNA. Samples collected in Cell-Free DNA BCT tubes are stable for up to 14 days at room temperature, allowing convenient sample collection, transport, and storage (Figure 1).

| Cell-Free DNA BCT tube specification |  |
| --- | --- |
| Blood Draw Volume | 10 mL |
| Anticoagulant | K_3_EDTA |
| Additive | Proprietary Stabilizing Agent |
| Storage Prior to use | Room Temperature |
| Shipment temperature | Room Temperature |


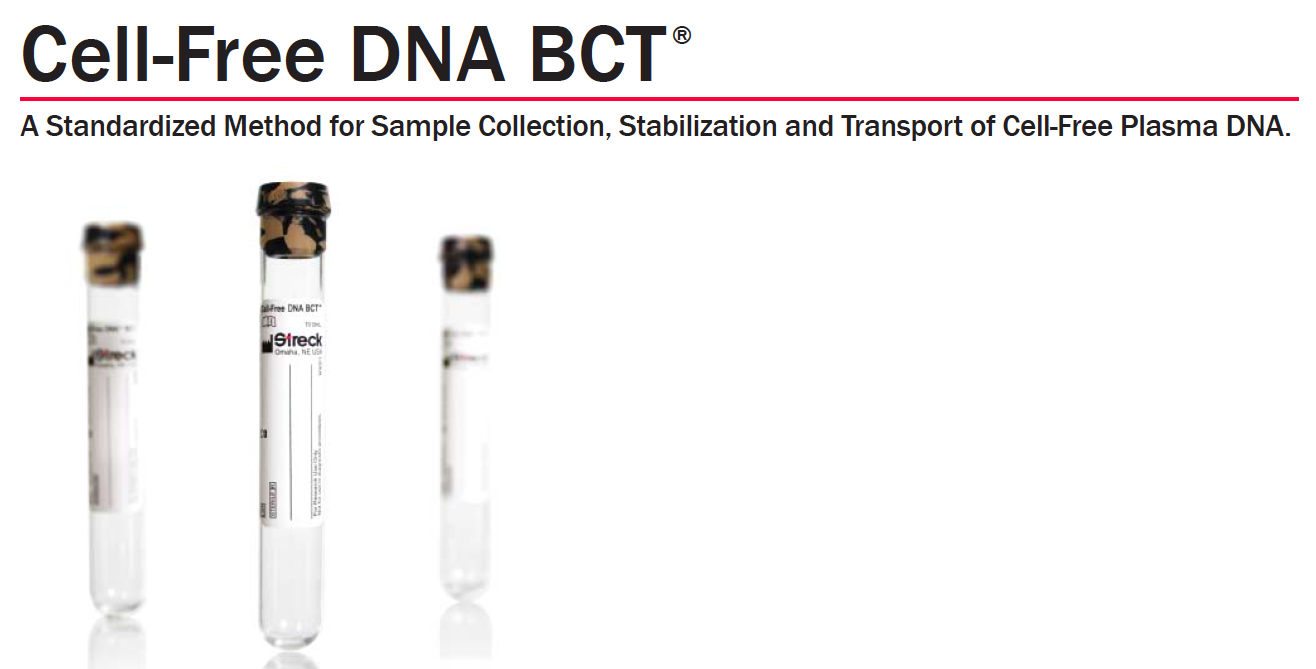


Figure 1. Cell-Free DNA BCT tube specification

Since Cell-Free DNA BCT tubes contain chemical additives, it is important to avoid possible backflow from the tube. To guard against backflow, take the following precautions:

- keep patient’s arm in the downward position during the collection procedure.
- hold the tube with the stopper in the uppermost position so that the tube contents do not touch the stopper or the end of the needle during sample collection.
- release tourniquet once blood starts to flow in the tube, or within 2 minutes of application. Fill tube completely.
- remove tube from adapter and immediately mix by gentle inversion 8 to 10 times. Inadequate or delayed mixing may result in inaccurate test results. One inversion is a complete turn of the wrist, 180 degrees, and back (Figure 2).

Figure 2. Cell-Free DNA BCT tubes after PB recovering

When stored at 18-30°C, unused Cell-Free DNA BCT tubes are stable through expiration date. Do not freeze unfilled Cell-Free DNA BCT tubes. Do not refrigerate or freeze blood collected in Cell-Free DNA BCT tubes.

Shipment of the BCT tubes to the reference centers must be carried out in adequate conditions because of their fragility.

APPENDIX B - Operative procedures for biological samples receipt and initial storage

1. Plasma selection from BCT or EDTA tubes

- Centrifuge tubes at 1300xg for 13 minutes at room temperature.
- Collect plasma in a separate 15 mL tube and centrifuge again at 1800xg for 10 minutes at room temperature.
- Store plasma at -80°C in 1mL aliquots.

1. ****Collection of B-cells from bone marrow or peripheral blood EDTA tubes****

Resuspend the bone marrow (BM) sample or the leftover blood from BCT in erythrocytes lysis buffer (NH_4_Cl) (1:5 dilution). The PB from EDTA tubes must be resuspended in erythrocytes lysis buffer (NH_4_Cl) (1:2 dilution). Leave 15 minutes at room temperature (lying flat at dark), then centrifuge 15 minutes at 450xg at room temperature. Discard the supernatant, resuspend the pellet in 10-15 mL of NH_4_Cl and centrifuge 10 minutes at 450xg at room temperature. Remove supernatant, resuspend in PBS or 0.9% NaCl (q.s.). Dispense 5-10x10^6^ cells in each tube, centrifuge 1 minute at 13000xg and discard supernatant. Cells can now be stored indefinitely, as dried pellets, at -80°C for further DNA extraction.

1. Cerebrospinal fluid storage

Dispense 1mL of cerebrospinal fluid (CSF) in each tube, centrifuge for 1 minute at 13000xg before ctDNA extraction.

APPENDIX C - Operative procedures for flow cytometry analysis

1. Screening panel

For screening purposes, BM samples from patients with an IgM monoclonal gammopathy should be processed and stained with at least an 8-color panel (detailed below). PB can also be analyzed in parallel. The screening consists in two 8-color tubes including the following monoclonal antibodies: 1) surface immunoglobulin-M (SIgM), CD25, CD22, CD19, CD20, CD45, CD38, and CD27; 2) intracytoplasmic IgM (CyIgM), kappa (CyIgκ) and lambda (CyIgλ), CD19, CD38, CD20, CD45, CD5. A minimum of 1 million cells should be acquired per tube.

| **Tube** | **FITC** | **PE** | **PERCP-Cy5.5** | **PE-Cy7** | **APC** | **APCH7/APCC7** | **PacB/BV450** | **PacO/OC515** |
| --- | --- | --- | --- | --- | --- | --- | --- | --- |
| 1 | SIgM  (5µL) | CD25  (10µL) | CD22  (10µL) | CD19  (5µL) | CD27  (5µL) | CD38  (3µL) | CD20  (5µL) | CD45  (5µL) |
| 2 | CyIgM  (5µL) | CyL  (5µL) | CD5  (10µL) | CD19  (5µL) | CyK  (5µL) | CD38  (3µL) | CD20  (5µL) | CD45  (5µL) |

1. Staining steps for surface membrane markers only

Calculate the total volume of surface membrane backbone (common) antibodies based on the number of tubes to stain and the specific titer for each one. Pipette these antibodies in one tube (backbone tube). Calculate the total volume of sample to be stained based on the number of tubes in the panel (100 µL/10 x 10^6^ per tube). Pipette this sample volume into the backbone tube. Mix well. Mix the correspondent amounts of the sample and backbone mix into the different tubes of the panel. Add the appropriate volume of other antibodies directed against cell surface markers (except for the backbone markers). If necessary, use PBS+0.5% BSA+0.09% NaN_3_ to reach a final volume of 200 µL per tube. Mix well. Incubate for 30 min at room temperature protected from light. Add 2 mL of 1X FACS Lysing Solution (10x FACS Lysing Solution diluted 1/10 vol/vol in dH_2_O; 10x FACS Lysing Solution from BD Bioscience, cat. Reference 349202). Mix well. Incubate for 10 minutes at room temperature protected from light. Centrifuge for 5 min at 540xg. Discard the supernatant using a Pasteur pipette or vacuum system without disturbing the cell pellet, leaving approximately 100 µL residual volume in each tube. Add 2 mL of PBS + 0.5% BSA+0.09% NaN_3_ to the cell pellet. Mix well. Centrifuge for 5 min at 540xg. Discard the supernatant using a Pasteur pipette or vacuum system without disturbing the cell pellet, leaving approximately 100 µL residual volume in each tube. Resuspend the cell pellet in 500 µL PBS+0.5% BSA (without NaN_3_).

1. Staining steps for combined intracellular and surface membrane markers

Add surface membrane antibodies in the appropriate volume (see panels). Mix well. Incubate for 20 min at room temperature protected from light. Add 2 mL of PBS + 0.5% BSA + 0.09% NaN_3_. Mix well. Centrifuge for 5 minutes at 540xg. Discard the supernatant without disturbing the cell pellet, leaving approximately 100 µL residual volume in each tube. Add 100 µL of Reagent A (fixative; Fix&Perm, An der Grub, Austria) and mix thoroughly. Incubate for 15 min at room temperature protected from light. Add 2 mL of PBS + 0.5% BSA + 0.09% NaN_3_ to the cell pellet. Mix well. Centrifuge for 5 minutes at 540xg. Discard the supernatant without disturbing the cell pellet, leaving approximately 100 µL residual volume in each tube, and resuspend the cell pellet by mixing thoroughly. Add 100 µL of Reagent B (permeabilizing solution; Fix&Perm), mix well and add the intracellular antibodies in the appropriate volume (see panel). Mix well. Incubate for 15 min at room temperature protected from light. Add 2 mL of PBS + 0.5% BSA + 0.09% NaN_3_ to the cell pellet. Mix well. Centrifuge for 5 minutes at 540xg Discard the supernatant without disturbing the cell pellet, leaving approximately 100 µL residual volume in each tube. Resuspend the cell pellet in 500 µL PBS + 0.5% BSA (without NaN_3_). Acquire the cells immediately after staining at medium flow rate or (if not immediately acquired) store at 4ºC (for 1 h maximum) until measured in the flow cytometer.

APPENDIX D - DNA extraction protocols for mutational studies

The following section will provide simplified protocols for gDNA and ctDNA extraction. Alternative methods can also be employed, provided they yield comparable results.

1. Example of gDNA extraction protocol (Maxwell® RSC Blood DNA- AS1400)

- Cell pellet needs to be thawed at room temperature prior to gDNA extraction.
- Resuspend 10x10^6^, 5x10^6^, <5x10^6^ pellet in 900, 600 and 300 µL of PBS or 0.9% NaCl, respectively.
- Take 300 µL of resuspended cells and add 300 µL of Lysis Buffer and 30 µL of Proteinase K
- Vortex 10 sec
- Incubate in heating/shaking block (56°C) for 20 mins
- Transfer to the cartridge prepared as required from the instrument
- Elute in 110 µL of Elution Buffer

1. Example of cfDNA extraction protocol (Maxwell® RSC LV ccfDNA kit-custom – AX1115)

- Plasma samples need to be thawed at room temperature prior to cfDNA extraction, and residual cellular debris removed using centrifugation at 13000xg for 3-5 minutes. In a 15 mL tube, add 1-4 mL of plasma an equal amount of Binding Buffer (AX301B) and 140µL of Resin (AX299) (resin must be shaken carefully before use).

Let shake for 45 min in a rotor

- Centrifuge at 1000xg for 2 min to pellet the resin. Remove the supernatant. If necessary, use a magnetic support to keep the magnetic beads on the bottom of the tube.
- Transfer the well #1 solution (the largest well in the cartridge) to the tube and mix gently to resuspend the resin. Then move back to the cartridge into the #1 well.
- Elute in 60 µL of buffer (MC139) and run the Maxwell RSC LV ccf DNA Custom Program
- To test the efficiency of fragmented DNA recovery, a panel of genomic reference loci (i.e., Albumin, RNAseP and ALU sequences) should be screened to assign cfDNA load (as suggested by Devonshire AS. [Anal Bioanal Chem](http://www.ncbi.nlm.nih.gov/pubmed/24853859) 2014).

APPENDIX E – ASqPCR protocol for *MYD88^L265P^* detection

A specific quantitative polymerase chain reaction (qPCR)–based allelic discrimination assay was developed for L265P mutation detection; the assay was designed with a single common forward primer and 2 reverse primers, based on the nucleotide difference at the 3’ terminal base (T or C). Thus, reverse primers differ in the nucleotide corresponding to the mutation, being specific either of the wild-type or the mutated allele; one TaqMan® probe is also necessary for the test. Each experiment requires two different PCR reactions: one for the detection of the mutation (with the mutated reverse primer) and the other one as a control of the DNA quality (using the wild-type reverse primer).

A simplified protocol including primers and probes and reaction setup is provided below, together with results analysis and interpretation.

1. Primers

- Forward: 5’-ACTTAGATGGGGGATGGCTG-3’
- Reverse wild-type: 5’-CCTTGTACTTGATGGGGATC**A**-3’
- Reverse mutated: 5’-CCTTGTACTTGATGGGGATG**G**-3’

1. Probe

5’-FAM-TTGAAGACTGGGCTTGTCCCACC-TAMRA-3’

1. Protocol

Two PCR reactions:

- 1. Detection of the mutation (total volume = 20 µL):

|  | Volume (per sample) | Final concentration |
| --- | --- | --- |
| H_2_O | 7.2 µL |  |
| Forward primer 18μM | 0.36 µL | 300 nM |
| Reverse mutated primer 18 μM | 0.36 µL | 300 nM |
| Probe 4 μM | 1.08 µL | 200 nM |
| TaqMan® Universal PCR Master Mix | 10.0 µL | 1X |
| DNA (20 ng/µL) | 1 µL | 20 ng |

- 1. Control of the DNA quality (total volume = 20 µL):

|  | Volume (per sample) | Final concentration |
| --- | --- | --- |
| H_2_O | 7.2 µL |  |
| Forward primer 18μM | 0.36 µL | 300 nM |
| Reverse wild-type primer 18 μM | 0.36 µL | 300 nM |
| Probe 4 μM | 1.08 µL | 200 nM |
| TaqMan® Universal PCR Master Mix | 10.0 µL | 1X |
| DNA 20 ng/µL | 1 µL | 20 ng |

Thermal cycling conditions: 10 minutes at 95°C, followed by 50 cycles of 95°C for 15 seconds and 60°C for 60 seconds in a StepOnePlus® Real-Time PCR (Applied Biosystems™).

1. Analysis and interpretation of the results

Once the PCR has finished, results are analyzed with the software of the equipment. Each sample will have a CT (cycle threshold) value (cycle in which fluorescence reaches 10-fold the basal emission and can be distinguished from the background noise), usually between 23 and 45 cycles. Samples are considered mutated if CT≤35 and the corresponding control (wild-type allele) has a CT value within the normal range. In addition, the difference between both CTs (∆CT = CTmutated - CTwild-type) must be ≤10.

APPENDIX F - ddPCR protocol for *MYD88^L265P^* detection

In the Pre-PCR zone, prepare the 20X primer and probe mix and the ddPCR reaction mix. One ddPCR master mix is created for each assay to perform 3 replicates per sample.

Prepare the ddPCR mix for a volume increased by 10% (for one replicate: 22 µL instead of 20 µL) to ensure having enough reaction mix volume for each replicate, and not to risk the generation of air bubbles into the DG8 cartridges, when loading the samples. Dispense the reaction mix in one well, based on the number of technical replicates needed, as described in the table below (i.e., for 1 replicate 16.5 µL and for 3 replicates 49.5 µL of mix).

In the DNA zone, load the corresponding amount of gDNA (20 ng/µL): for 3 replicates 49.5µL (16.5x3) of mix, add 16.5 µL (5.5x3) of gDNA. To ensure that the gDNA is thoroughly mixed, vortex, spin-down and then pipet the sample few times before adding the gDNA to the mix. Seal carefully the plate or the strips with optical adhesive film or caps, mix and spin down briefly. From this well, 20 µL of mix will be taken for droplet generation for each replicate.

Proceed with droplet generation, loading 20 µL of reaction mix and 60 µL of droplet generation oil into the proper DG8 cartridge wells.

It is recommended to:

- load the 20 µL of ddPCR mix in to the DG8 cartridge using a multichannel pipet with filter tips;
- pay attention when removing the DG8 gasket and always remove it from the NTC, or BC well position, to the positive control sample;
- always load the ddPCR reaction mix into the cartridge, before the oil;
- transfer the 40 µL of droplets/well to the hard-shell, high-profile, 96-well semi-skirted PCR plates using a multichannel pipet with no filter tips and immediately seal the wells with a scotch tape;
- load the technical replicates in different DG8 cartridges to avoid losing a full sample reaction due to a technical error that might happen during pipetting or failing during the droplet generation procedure;
- remove, carefully, any bubble created into the DG8 cartridge “sample” well during sample loading. If necessary, use a tip previously slightly soaked into the droplet generation oil.

**MYD88^L265P^ Assay CSTM DDPCR HEX/FAM ASSAY BIO-RAD**

- Forward (FP): 5'-CCTTGGCTTGCAGGT-3'
- Reverse (RP): 5'-TCTTTCTTCATTGCCTTGT-3'
- Probe for *MYD88^L265P^* MUT: **5'-**TGGGGATC**G**GTCGC-3' labeled with FAM
- Probe for *MYD88^L265P^* WT: 5'-TGGGGATC**A**GTCGCTT-3’ labeled with HEX

**ddPCR reaction Mix:**

| Reagents | 1 Reaction (µL) |  |  |
| --- | --- | --- | --- |
|  |  |  |  |
| 2X ddPCR Supermix for Probes (No dUTP) | 11 |  |  |
| 20X ASSAY MUT | 1.1 |  |  |
| 20X ASSAY WT | 1.1 |  |  |
| H2O | 3.3 |  |  |
| TOT mix | 16.5 |  |  |
|  |  |  |  |
| Amount of input gDNA (20 ng/µL) | 5.5 |  |  |
| TOT | 22 |  |  |
|  | | |  |
| MIX for 3 replicates | 49.5 µL |  |  |
| gDNA (100 ng) | 16.5 µL |  |  |
|  |  |  |  |
| Total Volume loaded for droplet generation | 20 µL |  |  |
| Total amount of droplet mix | 40 µL |  |  |

Thermal protocol:

95 °C  x 10 ‘

94 °C  x 30’’

x 40 cycles

55 °C  x  1’

98 °C x 10 ’

Note:

1) Each analysis must include:

- at least 2 replicates of POSITIVE CONTROL
- 3 replicates of each sample
- 3 replicates of WT control sample
- 3 replicates of no template control (NTC)

**Analysis and interpretation of the results:**

1. Only replicates with more (>=) than 9000 droplets must be considered for the analysis.
2. The threshold must be established manually, firstly checking the positive control and WT samples.
3. A threshold referred to the positive control and WT should be similar in all plates (std dev +/- 5%)
4. If no ch1 (blue dots) signals and only signal in ch1-ch2 (orange dots) is detected, this sample must be considered WT
5. If less than 3 positive signals are detected in ch1 (blue dots), verify the positivity by repeating the experiment. If data is confirmed (<3 blue dots), and ratio is below the cut-off ratio, sample must be considered WT.

APPENDIX G – ASqPCR protocols for *CXCR4^S338X^* (c.C1013G and c.C1013A) detection

Protocols, reaction setup and results analysis and interpretation are the same as for *MYD88^L265P^* mutations. They only differ in the primers and probes used.

1. CXCR4 C1013G
   1. Primers

- Forward: 5’-TTTCTTCCACTGTTGTCTGAACC-3’
- Reverse wild-type: 5’-GACTCAGACTCAGTGGAAACAGAT**G**-3’
- Reverse mutated: 5’-GACTCAGACTCAGTGGAAACAGAA**C**-3’
  1. Probe

5’-6FAM-TATGCTTTCCTTGGAGCCA-NFQ-MGB-3’

- 1. Protocol

Two PCR reactions:

Detection of the mutation (total volume = 20 µL):

|  | Volume (per sample) | Final concentration |
| --- | --- | --- |
| H_2_O | 7.2 µL |  |
| Forward primer 18μM | 0.36 µL | 300 nM |
| Reverse mutated primer 18 μM | 0.36 µL | 300 nM |
| Probe 4 μM | 1.08 µL | 200 nM |
| TaqMan® Universal PCR Master Mix | 10.0 µL | 1X |
| DNA (20 ng/µL) | 1 µL | 20 ng |

Control of the DNA quality (total volume = 20 µL):

|  | Volume (per sample) | Final concentration |
| --- | --- | --- |
| H_2_O | 7.2 µL |  |
| Forward primer 18μM | 0.36 µL | 300 nM |
| Reverse wild-type primer 18 μM | 0.36 µL | 300 nM |
| Probe 4 μM | 1.08 µL | 200 nM |
| TaqMan® Universal PCR Master Mix | 10.0 µL | 1X |
| DNA 20 ng/µL | 1 µL | 20 ng |

Thermal cycling conditions: 10 minutes at 95°C, followed by 50 cycles of 95°C for 15 seconds and 60°C for 60 seconds in a StepOnePlus® Real-Time PCR System (Applied Biosystems™).

1. CXCR4 C1013A
   1. Primers

- Forward: 5’-TTTCTTCCACTGTTGTCTGAACC-3’
- Reverse wild-type: 5’-GACTCAGACTCAGTGGAAACAGAT**G**-3’
- Reverse mutated: 5’-GACTCAGACTCAGTGGAAACAGTA**T**-3’
  1. Probe

5’-6FAM-TATGCTTTCCTTGGAGCCA-NFQ-MGB-3’

- 1. Protocol

Two PCR reactions:

Detection of the mutation (total volume = 20 µL):

|  | Volume (per sample) | Final concentration |
| --- | --- | --- |
| H_2_O | 7.2 µL |  |
| Forward primer 18μM | 0.36 µL | 300 nM |
| Reverse mutated primer 18 μM | 0.36 µL | 300 nM |
| Probe 4 μM | 1.08 µL | 200 nM |
| TaqMan® Universal PCR Master Mix | 10.0 µL | 1X |
| DNA (20 ng/µL) | 1 µL | 20 ng |

Control of the DNA quality (total volume = 20 µL):

|  | Volume (per sample) | Final concentration |
| --- | --- | --- |
| H_2_O | 7.2 µL |  |
| Forward primer 18μM | 0.36 µL | 300 nM |
| Reverse wild-type primer 18 μM | 0.36 µL | 300 nM |
| Probe 4 μM | 1.08 µL | 200 nM |
| TaqMan® Universal PCR Master Mix | 10.0 µL | 1X |
| DNA 20 ng/µL | 1 µL | 20 ng |

Thermal cycling conditions: 10 minutes at 95°C, followed by 50 cycles of 95°C for 15 seconds and 60°C for 60 seconds in a StepOnePlus® Real-Time PCR System (Applied Biosystems™).

**APPENDIX H – Sanger sequencing protocol for *CXCR4^WHIM^***

1. Primers

- Forward: 5’-GCTGCCTTACTACATTGGGATCAGC-3’
- Reverse: 5’-TTGGCCACAGGTCCTGCCTAGACA-3’

1. Protocol

|  | Volume (per sample) | Final concentration |
| --- | --- | --- |
| H_2_O | 18.05 µL |  |
| Forward primer 10μM | 0.5 µL | 0.2 μM |
| Reverse primer 10 μM | 0.5 µL | 0.2 μM |
| Buffer | 2.5 µL | 1x |
| MgCl_2_ | 2 µL | 2 mM |
| dNTPs | 0.2 µL | 0.1 mM |
| Taq Polymerase | 0.25 µL | 0.05 U/µL |
| DNA (50 ng/µL) | 1 µL | 50-100 ng |

Thermal cycling conditions: 10 minutes at 94°C, followed by 30 cycles of 94°C for 45 seconds, 65ºC for 45 seconds and 72°C for 60 seconds, and a final step of 5 minutes at 72ºC.

PCR product is then purified and used in the sequencing reactions. Each sample must be sequenced in both directions (i.e., from the forward primer and from the reverse primer). Sequencing primers are the same used for the PCR reaction. Purification and sequencing protocols can be specific to each laboratory.

APPENDIX I - FISH analysis for 6q deletion (del6q)

6q21 deletion in WM is screened using a centromeric probe for chromosome 6 CEP 6 (Abbott Laboratories). A minimum of 100 cells are analyzed using Vysis scoring criteria.

To perform the test, slides are prepared according to the standard procedure and fixer is removed as needed to have an adequate concentration of cells per 15 µL in the center of the slides.

Place slides in pepsin solution (10 mL HCl 0.1 N or 5 mL HCl 0.2N + 90 mL distilled water + 200 µL pepsin) and incubate in a shaking water bath at 37ºC for 5 minutes. If using a 50 µL aliquot of pepsin (instead of 200 µL), incubation time must be 10 minutes. Transfer the slides to a container with 0.5x SSC/Tween 20 (975 mL distilled water + 25 mL 20x standard saline citrate, SSC, + 100 µL Tween 20) and incubate for 20 minutes in a shaking water bath at 37ºC. Subsequently, dehydrate slides in 70%, 85% and 100% ethanol for 2 minutes each at room temperature while shaking, and air dry. Apply 5 µL of the probe* diluted 1/50 in amplification buffer (Insitus Biotechnologies®) to the slides, cover them with an 18x18 coverslip and seal with rubber cement.

The next day wash the slides first with 0.4x SSC/Tween 20 (980 mL distilled water + 20 mL 20x SSC + 3 mL Tween 20) for 2 minutes in a shaking water bath at 73ºC, and then, with DAPI solution (70 mL 2x SSC + 70 µL DAPI, freshly made) for 2 minutes. Add a drop of Vectashield® to the slide and apply a 24x50 coverslip. Finally, seal the slides with transparent nail polish and read in the fluorescence microscope.

The cut-off point for the identification of the del6q alteration is set at ≥10% cells with abnormal signal.
